# Accelerated Miocene incision along the Yangtze River driven by headward drainage basin expansion

**DOI:** 10.1126/sciadv.adh1636

**Published:** 2023-09-08

**Authors:** Alexander Rohrmann, Eric Kirby, Wolfgang Schwanghart

**Affiliations:** ^1^ Institute of Geological Sciences, Freie Universität Berlin, Berlin, Germany.; ^2^ Department of Earth, Marine, and Environmental Sciences, University of North Carolina, Chapel Hill, Chapel Hill, NC, USA.; ^3^ Institute of Environmental Science and Geography, University of Potsdam, 14476 Potsdam-Golm, Germany.

## Abstract

Along the southeastern margin of the Tibetan Plateau, the onset of rapid fluvial incision during the Miocene is commonly attributed to growth of high topography. Recent recognition of lacustrine strata preserved atop interfluves, however, suggest that headward expansion of river networks drove migration of the topographic divide. Here, we explore the impact of this process on fluvial incision along the Yangtze River. Landscape evolution simulations demonstrate that expansion of the Yangtze watershed since the Late Miocene could be responsible for 1 to 2 kilometers of fluvial incision. The distribution of modern knickpoints and river profiles is consistent with this hypothesis. We suggest that increased erosive power associated with capture and basin integration drove accelerated incision during the Late Miocene. Our results imply that eastern Tibet was elevated before middle Cenozoic time and that the tempo of fluvial incision may be out of phase with uplift of plateau topography.

## Teaser

Miocene headward drainage basin expansion drove incision along the Yangtze River in eastern Tibet.

## INTRODUCTION

The presence of extensive low-relief landscapes perched above deeply incised canyons has, for decades, been considered to represent relict topography elevated during orogenesis (*1*). Hence, fluvial incision into these often long-lived landscapes (*2*) affords insight into the timing, rate, and patterns of rock and surface uplift along the margins of orogenic plateaus (*3*). However, the pace and tempo of fluvial incision may be strongly modulated by other factors including climatically forced changes in transport capacity and erosive efficiency (*4*, *5*), drainage divide migration leading to expansion and contraction of drainage basin area (*6*–*8*), autogenic feedbacks between river incision and landsliding (*9*), and basin integration via drainage capture (*10*). Deconvolving the effects of these various mechanisms, and the degree to which they promote or retard fluvial incision, is critical for interpretations that relate the timing and rates of incision to surface uplift (*3*).

**Fig. 1.**
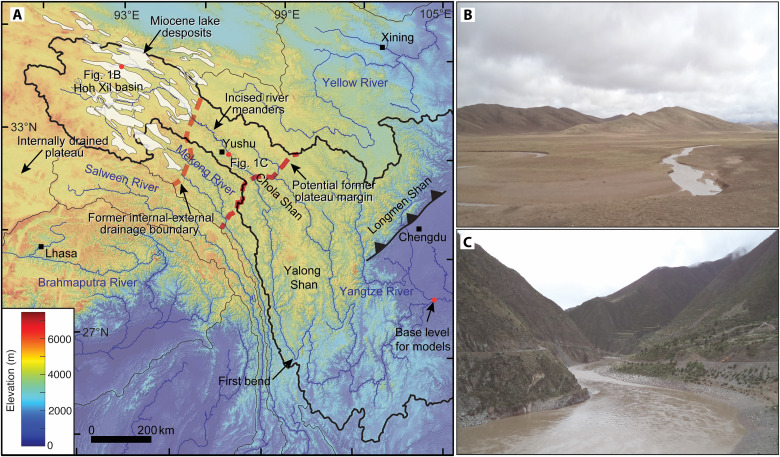
Overview of eastern Tibet with major river catchments and distribution of Miocene lake sediments. (**A**) Digital elevation map (90 m) of eastern Tibet with catchment areas of the Yellow, Yangtze, Mekong, Salween, and Brahmaputra River. The extent of Miocene lake deposits in (*27*) is highlighted in white polygons, and dashed red and orange lines mark the western former internal drainage boundary and a more easterly potential former plateau margin. (**B**) Photo illustrates the originally preserved upstream low-relief, high-elevation landscape in eastern Tibet with river meanders. (**C**) Incised meander belt near Yushu forming up to 2 km of relief.

This problem is perhaps most clearly manifested in ongoing debates over the geomorphic evolution of the southeastern Tibetan Plateau. Here, the Salween, Mekong, and Yangtze rivers descend from the low-relief plateau and flow across the topographic margin of the plateau through deeply incised gorges (Fig. 1). The presence of low-relief landscapes perched on interfluves up to ~4 km above the modern rivers (*11*), coupled with thermochronologic records of rapid exhumation during Late Miocene time (*3*, *12*–*14*), led to the suggestion that these landscapes are relict remnants of a once extensive, low-relief landscape that has been elevated during plateau growth. Recently, however, these interpretations have been called into question. First, it has been suggested that the low-relief surface of the plateau actually formed in situ, as a consequence of drainage divide migration (*15*). Although this mechanism has subsequently been argued to be inconsistent with the geomorphology of the region (*16*), the time-space patterns of fluvial incision remain poorly known along these rivers (*17*). More recently, it has been pointed out that the low-relief surface of the plateau is out of equilibrium with the spatial uplift rate, and therefore, the formation of this landscape at high or low elevation cannot be deduced from the landscape itself (*18*). Despite this finding, recent efforts suggest that low-relief surfaces may evolve in response to simple eastward propagation of plateau uplift and associated localized fluvial incision (*19*). Second, paleoaltimetry from tertiary sedimentary basins in the region (*20*) suggests that modern elevations may have been established by Eocene-Oligocene time, consistent with thermochronologic data from the central Tibetan Plateau (*21*). Recent attempts to reconcile accelerated exhumation along the Mekong River during Miocene time appeal to climatically drive increases in discharge (*22*). However, this interpretation has been questioned, and explanations involving a multiphase tectonic history (*23*) or activity along a deep crustal ramp (*24*) have been proposed as alternative explanations. Last, although headward erosion into the plateau margin along the Yellow River (Fig. 1A) has been argued to explain the time lag between rapid quaternary incision (*25*) and mountain building in northeastern Tibet, the role of headward basin integration is unknown along major rivers in southeastern Tibet. All currently proposed hypotheses for the formation mechanism of the plateau’s low-relief high-elevation landscape and its fluvial incision history consider that the watershed drainage area remained static throughout the plateau’s evolution. Here, we evaluate the potential impact of notable changes in drainage basin extent over time.

### Evidence for river network expansion in eastern Tibet

**Fig. 2.**
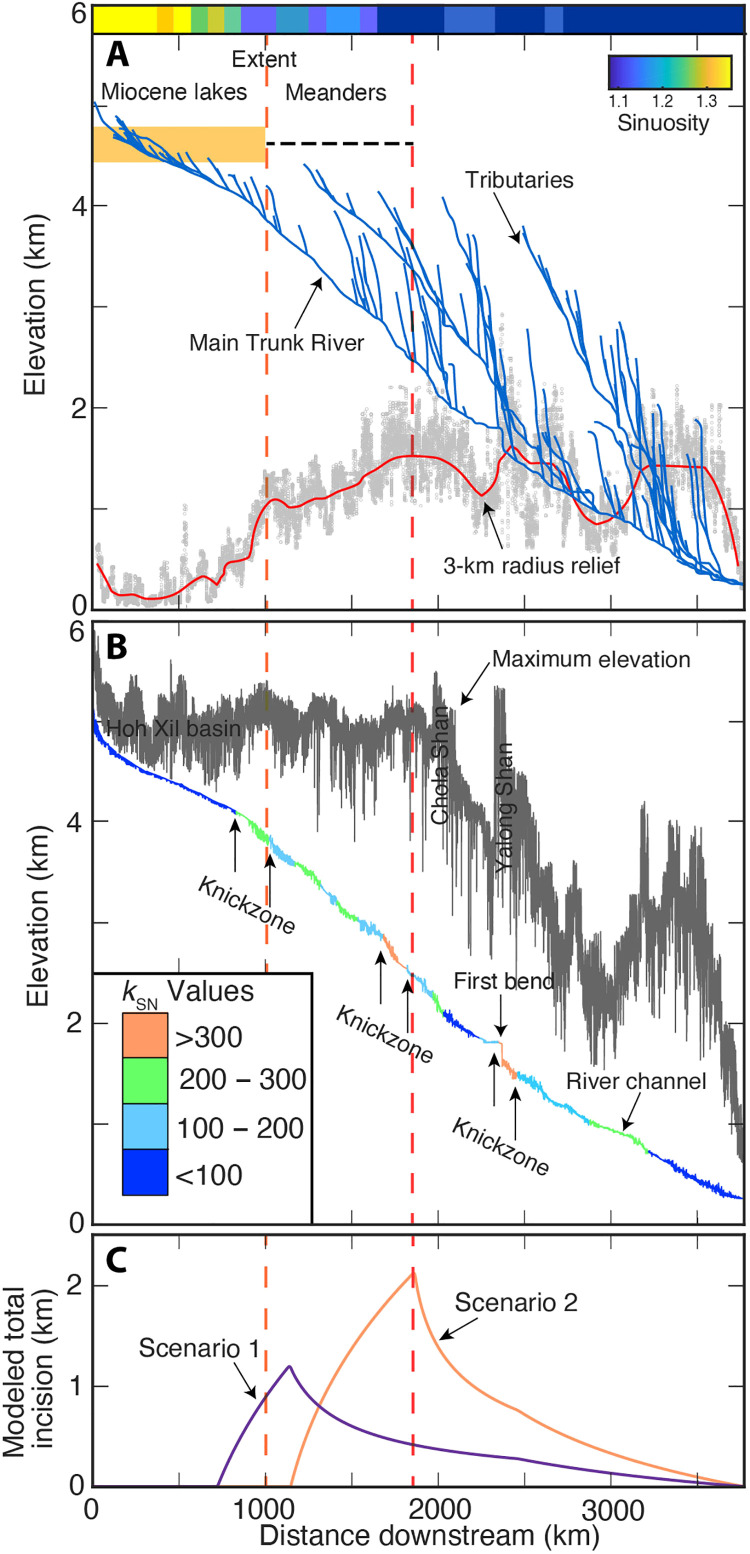
Geomorphology, low-relief, high-elevation surface and 1D LEMs results of the Yangtze River. (**A**) Tributaries and main trunk of the Yangtze with the extent of Miocene lake deposits (*27*) (shown as an orange box) and mapped extent of meandering river stretches (shown as a dotted black line). The orange box represents the mapped vertical and lateral extent of Miocene lake sediments. In addition, the degree of river sinuosity along the Yangtze is shown. Note the high river sinuosity for the upper-most Yangtze catchment. The red line in the lower part of the figure represents the mean 3-km radius relief along the Yangtze taken from the 90-m digital elevation model (single data points are shown in the background as a gray point cloud). Note the rapid increase in the 3-km radius relief from around 200 m to more than 1000 m that coincides with the easternmost extent of Miocene lake sediments. (**B**) A 40-km maximum and minimum elevation swath profile along the Yangtze. The minimum elevation corresponds to the Yangtze that serves as a regional base leve,l and the maximum elevation coincides for ca. 2000 km downstream with the low-relief, high-elevation landscape. Channel steepness (*k*_sn_) values are superimposed onto the minimum elevation, and knickzones are highlighted in the figure. The knickzones generally correspond to major boundaries in the extent of the Miocene lake sediments and meandering river stretches. (**C**) 1D LEMs incision results of the Yangtze River for the two scenarios considered [(i) purple: extent of lakes; (ii) orange: extent of lakes and meanders) modeled with the 1D LEM (*36*). Modeled total incision along the Yangtze, i.e., initial stream profile minus final stream profile.

The evolution of landscapes in response to an increase in rock uplift rate or base-level fall is reasonably well understood (*16*, *17*, *26*), although recent studies highlight the potential complexities associated with drainage reorganization during sustained uplift of an active mountain range (*7*, *8*). The headwater regions of the Yangtze River (locally known as the Jinsha River) in eastern Tibet provide the unique opportunity to study the effect of a substantial upstream-river network expansion and increase in fluvial runoff. Here, Early to Middle Miocene lake sediments (Wudaoliang Group and related strata) (*27*) are preserved across an extensive region of central and eastern Tibet (Fig. 1A). These strata are <300 m thick and were deposited in lake and lake-marginal environments between ~25 and 13 million years (Ma) ago (*27*). In the central Hoh Xil basin, these strata lie with angular unconformity above folded Eocene-Oligocene strata of the Fenghuoshan Group (*27*–*29*). They are interpreted to have been deposited an extensive lake system (*27*) at relatively high elevation of >3 to 4 km (*30*), although these elevation estimates have been challenged (Fig. 1A) (*31*). Along the headwater reaches of the Yangtze and Mekong rivers, Wudaoliang Group strata are now preserved on interfluves ~100 to 300 m above the modern drainage network (Figs. 1A and 2A), implying that the drainage basin area of the Yangtze River has expanded subsequent to mid-Miocene time.

Although the easternmost extent of Miocene lake sediments represents an unambiguous marker for the position of a former topographic divide between internal and external drainage in eastern Tibet, several lines of evidence suggest that a topographic divide representing a former plateau margin may have been located up to several hundred kilometers farther east (Fig. 1A). First, numerous workers have noted the unusually narrow shape of the watersheds in the three rivers’ region (*32*); in the Yangtze watershed, this narrow “neck” coincides with a belt of incised meanders along the river (Figs. 1, A and B, and 2A). The planform geometry of these meanders typically forms in a low-relief, low-gradient landscape and becomes entrenched upon the onset of rapid incision (*33*). Second, this incised meander belt coincides with the eastward extent of preserved low-relief, high-elevation surfaces that appear to be on grade with the internally drained upland area hosting Miocene lakes (Fig. 2, A and B). Last, recent thermochronologic data from the Yalong Shan in southeastern Tibet (*34*) and the Longmen Shan in eastern Tibet (*35*) (Fig. 1A) suggest that rapid cooling initiated in Eocene-Oligocene time, presumably marking the onset of mountain building along the margin of the plateau (*34*). Although this thrust belt is located well to the east of the inferred topographic margin, the timing of exhumation suggests that the plateau in this region likely began experiencing surface uplift early in Cenozoic time and might have ceased by no later than ~10 Ma ago (*24*). Thus, it seems reasonable to presume that the former topographic divide between internal and external drainage was located up to ~700 km east of the easternmost relict Miocene lake deposits and includes the region of preserved low-relief surfaces and associated meander belts (Figs. 1A and 2). Relative to the catchment upstream of local base level in the Sichuan Basin (Fig. 1A), these boundaries imply that the Yangtze River watershed expanded by >25 to 40% (>1000 to 1725 km of additional channel length) at some point since the cessation of lacustrine deposition around 13 to 15 Ma ago.

## RESULTS

Here, we evaluate the consequences of integrating these internally drained lacustrine basins into the Yangtze River catchment for fluvial incision both upstream and downstream of the point of capture (Figs. 1A and 2B). We conduct one-dimensional (1D) and 2D landscape evolution modeling (LEM) using a stream-power type rule (*36*, *37*) to characterize the response of the Yangtze River and its tributaries to an increase in upstream drainage area. We consider two scenarios in a 1D LEM that represent adding ~1000 and ~1725 km of channel length, respectively, or 0.85 × 10^6^ and 1.36 × 10^6^ m^2^ of previously internally drainage basin area (Fig. 2C and fig. S1) (for details see Material and Methods). In the 2D LEM, we explore whether the spatial distribution of knickpoints in the Yangtze basin is consistent with incision in response to basin expansion (for details see Material and Methods). We compare our results with analysis of river longitudinal profiles and the spatial distribution of knickpoints in the proposed region of integration.

### 1D landscape evolution model results

The pattern of response in the 1D LEM is similar in both scenarios considered here (for details see Material and Methods); reaches upstream of the breached divide experience rapid incision associated with the development and migration of a distinct knickpoint, while reaches downstream experience a pulse of incision associated with the instantaneous disequilibrium between uplift rate and incision rate [cf. (*8*)] that decays in a downstream direction (Fig. 2C and fig. S1). For the first scenario, where the internal/external drainage boundary is taken to be the easternmost extent of Miocene lacustrine deposits, expansion of the watershed over the last 15 Ma is associated with the following characteristics (Fig. 2C and fig. S1): (i) establishment of a distinct knickpoint that retreats ~525 km at a constant rate through time of 35 km/Ma; (ii) maximum vertical incision of ~1.2 km localized at the point of initial breaching; and (ii) depth of incision decreases downstream but notably remains >500 m for at least ~1000 km downstream of the capture. For the second scenario, where the eastern location of the breached drainage divide sits outboard of the belt of incised meanders, these characteristics scale with the size of the additional drainage area: (i) The knickpoint migrated ~665 km at a constant rate through time of ~44 km/Ma; (ii) maximum vertical incision of ~2.1 km occurs at the point of the breached boundary; and (iii) incision downstream of this point exceeds 1.0 km for ~1000 km downstream of the capture point (Fig. 2C and fig. S1). In general, we do not expect a perfect match of the 1D LEM versus the actual stream profile, as they are subject to a set of simplifying assumptions: (i) The initial profile is in steady state with spatially uniform rock uplift rate; (ii) the erodibility (*K*) of the underlying rock is uniform across the catchment; (iii) all channel reaches respond to the increase in discharge due to the increase in upstream river-network expansion. In most landscapes, one or more of these assumptions will be violated to some degree, resulting in variability in the distribution of knickpoints (*38*) and the shape of the modeled stream profile versus observed. A further limitation of the 1D LEM is that there is no communication of accelerated incision to stream segments upstream of knickpoints that have been observed on smaller scales [e.g., (*39*)]. The incision is strictly limited to the region downstream of the retreating knickpoint, as the area added above the knickpoint is progressively uplifted. The 1D LEM is therefore not able to capture the observed modern incision of up to 300 m into the Miocene lake sediments that were part of the low-relief, high-elevation surface (Figs. 1A and 2B) (*27*). Despite these limitations, the 1D LEM is able to capture the substantial effects of drainage basin expansion to downstream incision below the retreating knickpoint within the Yangtze.

### 2D landscape evolution model results

**Fig. 3.**
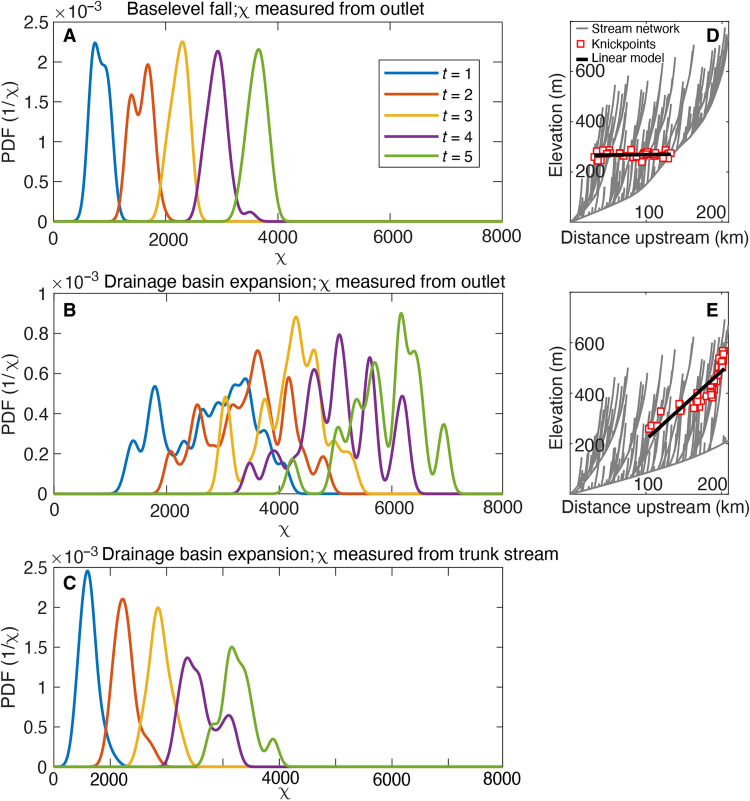
2D LEM nonparametric estimation of the dependence of knickpoints on χ and simulated vertical distribution of knickpoints. (**A** to **C**) Probability density functions of knickpoints in χ space with different scenarios: (A) base-level fall/uplift rate change measured from outlet, (B) drainage basin expansion measured from outlet, and (C) drainage expansion measured from trunk stream. (**D**) Simulated vertical distribution of knickpoint locations as a response to base-level fall/change in uplift rate. (**E**) Simulated vertical distribution of knickpoint locations as a response to drainage basin expansion. PDF, probability density function.

**Fig. 4.**
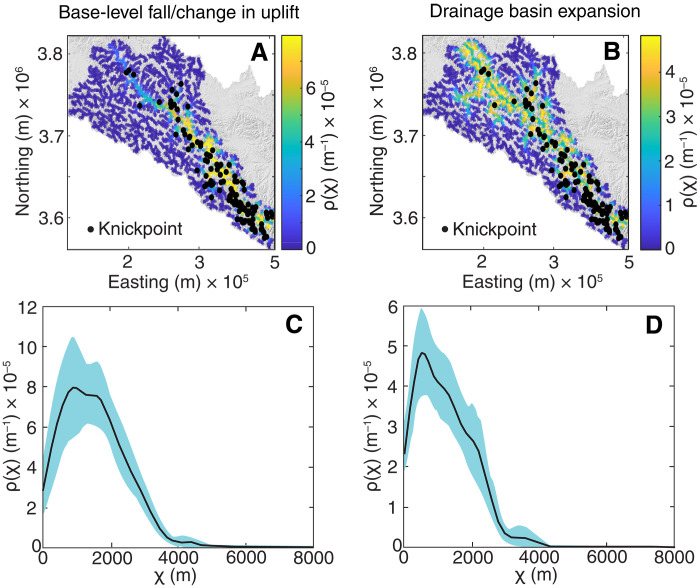
Distribution of observed knickpoints within the upper Yangtze and spatial probability distribution of simulated knickpoints in χ space. Map view of observed actual knickpoints (black points) and simulated spatial probability distribution of knickpoint locations (color bar) in χ space for the 2D LEM scenarios (**A**) base-level fall/change in uplift measured from the base-level and (**B**) drainage basin expansion measured from the trunk river. Plot of the observed actual Yangtze knickpoint distribution with the 2D LEM scenarios in χ space (**C**) for base-level fall/change in uplift and (**D**) accelerated incision due to drainage basin expansion.

Simulations with 2D LEM [TopoToolbox Landscape Evolution Model (TTLEM)] (*37*) demonstrate that the vertical and horizontal pattern of knickpoints expected during a transient response to an increase in catchment area differs from that expected due to an increase in uplift or base-level fall (Figs. 3 and 4 and figs. S2 and S3) (for details, see Material and Methods). As has been previously shown (*26*), an increase in uplift rate generates knickpoints that migrate upstream the trunk river and successively into tributaries. Drainage basin integration, in contrast, leads to coeval incision along the entire trunk river, albeit at different rates, that represents a simultaneous drop of the erosional base of all tributaries. These different responses can be identified using χ analysis (*40*) of the network distribution of knickpoints. In the case of a transient response to a base-level fall or increase in uplift rate, knickpoints are distributed systematically at χ values determined relative to the base level (Fig. 3A), whereas in response to basin integration, knickpoints are widely distributed throughout the basin (Fig. 3B). However, when the χ values are recalculated relative to the tributary confluences with the trunk river (Fig. 3C), knickpoints are systematically associated with values of χ. Similarly, the vertical distribution of knickpoints is different between models. In response to an increase in uplift rate, knickpoints are predicted to cluster around a specific elevation (*16*), whereas in response to an increase in drainage area, knickpoint elevations should be parallel to the longitudinal profile of the trunk river (Fig. 3, D and E). These characteristic differences in the association of knickpoints and channel network suggest a potentially diagnostic mechanism to identify knickpoints associated with capture and increases in drainage basin area.

## DISCUSSION

### Comparison of modeling results and observations in the Yangtze watershed

To evaluate how the incorporation of previously internally drained regions of the upper Yangtze watershed may have influenced the incision history of the Yangtze River (Figs. 2 to 4), we compare our 1D and 2D LEM results with the horizontal and vertical distribution of knickpoints on tributaries of the Yangtze, as well as the shape of the trunk profile itself. This comparison implicitly assumes that knickpoint migration rates are a function of stream power (*40*). We also compare the depth of incision with other geologic and geomorphic observations within the catchment, such as the location of the low-relief, high-elevation surfaces and Miocene lacustrine sediments.

The extent of the low-relief, high-elevation surface in the vicinity of the Yangtze is best illustrated by visualizing the topographic relief in a 3-km radius (Fig. 2A) and by maximum and minimum elevations along a swath profile centered along the river (Fig. 2B). The topographic relief along most of the upstream reaches averages ~200 m (Fig. 2A) but abruptly rises to values above ~1000 m near the belt of incised meanders (Fig. 2A); this increase also coincides with the easternmost extent of the Miocene lake sediments preserved atop the plateau surface (*27*). The presence of the Miocene lacustrine sediments implies that the low-relief, high-elevation surface must have been established before upstream drainage basin expansion. The region of elevated relief also corresponds to a distinct knickzone along the upper Yangtze (Fig. 2B) that marks a transition from the relict low-relief landscape to the more dissected reaches of the river. Along this reach of the river, topographic relief continues to increase from 1000 m to greater than 1700 m coincident with the steepest reaches of the Yangtze River; channel steepness (*k*_sn_) values along this reach exceed 300 m^0.9^ (Fig. 2B). This steepened reach also delimits the easternmost extent of the belt of incised meanders and of the low-relief, high-elevation surfaces (Fig. 2B). Farther downstream from this point, the maximum elevation decreases, with a few localized topographic highs associated with the Chola and Yalong Shan massifs (Fig. 1A). Collectively, these observations support the contention that the easternmost extent of the meandering river stretches and that the extent of the low-relief, high-elevation surface represent the location of a potential former plateau margin that also served as the divide between former internally and externally drained basins (Fig. 1A).

Our 1D river profile model is able to simulate the development of topographic relief along the Yangtze during 15 Ma of incision entirely as a consequence of upstream drainage integration (Fig. 2C and fig. S1). The horizontal and vertical distributions of tributary knickpoints straddling the upper Yangtze also appear to be the consequence of incision driven by capture of upstream basin area, as knickpoints are present at systematically higher elevations upstream, forming an array that parallels the channel profile. As noted above, knickpoints formed during base-level fall or a change in uplift rate should cluster along the same elevation band (Fig. 3D), whereas knickpoints formed during incision driven by headward capture of drainage area should progressively increase in elevation upstream (Fig. 3E). Comparison of the distribution of observed knickpoints of the Yangtze with the location of predicted knickpoints by each of the 2D LEM scenarios of either base-level fall/change in uplift measured from the base-level (Fig. 4A) or drainage basin expansion measured from the trunk channel (Fig. 4B) in χ space further confirms this inference. Here, the scenario of a simultaneous drop of the erosional base level for each tributary due to drainage basin expansion generates a unimodal distribution and a tighter fit (Fig. 4D), whereas the scenario of a base-level fall/change in uplift rate generates a wider distribution (Fig. 4C).

Although both scenarios appear to explain the position of knickpoints in the lower reaches of the Yangtze River, the position of observed knickpoints in the upper reaches of the Yangtze, above the point of capture, is only correctly predicted by the 2D LEM scenario of drainage basin expansion measured from the trunk river (Fig. 4, B and D). These results thus provide compelling evidence that recent incision along the central reaches of the Yangtze River occurred in response to an increase in upstream drainage basin area. Furthermore, our results suggest that analysis of knickpoints in χ space can be a useful discriminant to evaluate drivers of transient fluvial incision in other landscapes worldwide.

### Implications for fluvial incision in eastern Tibet

The canonical interpretation of the low-relief, high-elevation surfaces in eastern Tibet suggests that these are remnants of a formerly continuous, low-relief landscape that is undergoing progressive fluvial dissection in response to surface uplift of the plateau (*3*). These studies point to approximately 10-fold difference in both short-term erosion rates (10^5^ years) and long-term exhumation rates (10^6^ years) on low-relief surfaces and in deeply incised canyons (*3*, *14*). However, recent studies suggest that low-relief surfaces might result from the internal dynamics of divide migration and river capture during sustained uplift (*15*). Our analysis of knickpoints and channel profiles along the Yangtze River is not consistent with the expectations of river network disruptions as envisioned by this study (*15*). Rather, the extent of Miocene lake deposits (*27*) and incised meandering river stretches (Figs. 1A and 2A) indicates that isolated patches of low-relief, high-elevation surface were indeed once a continuous landscape that contiguous with the internally drained high plateau (*8*, *11*). The presence of endorheic conditions in the Wudaoliang Formation (*27*) suggests that the plateau surface had developed its low relief before the Early Miocene, and subsequent integration of these closed drainage basins drove accelerated incision of 1 to 2 km by increasing discharge and stream power along the trunk channel (Figs. 2C and 4). Although our study supports previous interpretations that the landscape in eastern Tibet is undergoing transient fluvial dissection in response to uplift of the plateau surface [e.g., (*3*, *19*)], we suggest that recent accelerations in incision are a consequence of breaching of the topographic divide that separated the internally drained plateau from its periphery. Thus, the timing and rates of incision along trunk channels such as the Yangtze River likely do not provide a direct constraint on the timing of plateau uplift. This highlights the fact that the Tibetan landscape is a nonunique landscape and that topography alone to derive geodynamic processes in space and time is an ill-posed problem (*18*). Moreover, our analysis suggests that changes in the amount of fluvial run-off due to periods of increased monsoonal precipitation (*22*) are not required to drive accelerated incision. Although the timing of lake recession is not yet well known, it seems likely that breaching of the drainage basin divide may have played a role in the demise of these extensive lake systems. Such a scenario could potentially trigger pulsed episodes of rapid incision if fill-and-spill dynamics connected previously closed basins across large areas of the plateau (*25*).

In general, the timing of drainage basin expansion after 13 to 15 Ma ago and the consequent downstream incision of ~1 to 2 km overlap in time with the formation of deep canyon incision in southeastern Tibet. The onset of this deep canyon formation ranges between ca. 10 and 20 Ma based on apatite-helium and fission-track low-temperature thermochronology [e.g., (*11*, *14*)]. The amount of downstream incision due to drainage basin expansion is not sufficient to explain the total exhumation of 2 to 4 km recorded by low-temperature thermochronometers. However, it is intriguing that even the simple 1D profile models suggest that at least half of this incision might result from drainage basin expansion. This is corroborated by pulsed incision and ca. 1 km of canyon downcutting between 15 and 10 Ma ago at the first bend region of the Yangtze (*41*). The timing of this incision is in line with the time of upstream drainage basin expansion starting at 15 Ma ago. The increase in discharge due to drainage basin expansion would result in simultaneous incision and downcutting along the entire Yangtze (trunk river) as opposed to a solitary wave of incision migrating upstream. Thus, we conclude that the carving of deep canyons in southeastern Tibet was, at least in part, a consequence of drainage basin expansion.

One implication of our results pertains to the exhumation history of the plateau upstream of the breached divide. In this region, the only available thermochronology data are (U-Th)/He ages from detrital samples of modern river sediment (*42*). Populations of individual grain ages have a significant component that is younger than 20 Ma, and some ages are as young as 1 to 4 Ma (*42*). Because the integration of the upper Yangtze watershed did not take place until after 13 to 15 Ma ago, many of the tributary watersheds where these samples were collected were not integrated into the externally drained margin of the plateau until after this time. Therefore, our findings are at odds with the interpretation that the young thermochronologic ages reflect an increase in uplift rate within the upper reaches of the Yangtze and Mekong during the Late Miocene (*42*). Rather, it appears that these portions of the drainage basin would have been isolated from the effects of relative base level fall at the plateau margin until after breaching of the former divide. Future work is required to determine whether these Miocene and Pliocene cooling ages represent a population derived from young volcanic rocks, outliers in the apatite-helium age distribution, or may have been derived from localized region of rapid uplift, where the amount of exhumation exceeds the present-day topographic relief.

Our results also carry implications for the provenance of fluvial sediments interpreted to record the history of erosion along the Yangtze River system. The formation of the Yangtze and its former extent remains a topic of active inquiry. On the basis of the provenance of fluvial sediments preserved in depocenters in eastern Tibet, workers have suggested that the inception of this transcontinental rivers system occurred anywhere from late Cretaceous to Holocene [e.g., (*43*–*46*)]. Our findings are consistent with the notion that the connection of the uppermost river reaches (north and west of the modern city of Yushu; Fig. 1) occurred at some point after 13 to 15 Ma ago. Therefore, our study sets an important maximum bound on the expectation for when detritus with provenance from the upper watershed would be expected to have reached depocenters downstream. Before cessation of Miocene lacustrine deposition in headwater reaches, material from the Tibetan Plateau would not have been available to downstream basins. Moreover, our results suggest that an abrupt change in the provenance of sediment would have occurred during drainage basin expansion, as opposed to a potentially more gradual change in response to progressive uplift and relative base-level call (*24*).

Last, our results also bear on models that consider the drainage basins of the Salween, Mekong, and Yangtze rivers to have been distorted during distributed shear (*19*, *32*) and/or extrusion in eastern Tibet. Both of these interpretations presume that the initial geometry of drainage basins was established before Indo-Asia collision. Our data suggest that the unusual basin geometries are, in fact, a consequence of relatively recent expansion of the headwater regions of these continental-scale rivers into an internally drained plateau. Thus, their use as a finite strain marker (*32*) is suspect. Our analysis further suggests that the former divide between the internally and externally drained regions of the plateau may have once sat in a position intermediate between its present-day location (Fig. 1A) and the regions of deep Cenozoic exhumation that mark the eastern margin of the plateau (*34*). We note that recent work suggests that local variations in uplift rate, as exemplified along the Mekong River (*23*), may make it difficult to uniquely identify the former position(s) of the drainage divide.

In summary, our study offers a partial answer to a long-lived and, at times fierce, debate over the timing and mechanism(s) by which deeply incised canyons along the Yangtze, Mekong, and Salween rivers were carved in eastern Tibet. Although the precise timing remains uncertain, our analysis reveals that expansion of the Yangtze network into a previously internally drained plateau could have driven ~1 to 2 km of downstream river incision, even in the absence of changes in climate (*22*) or tectonics (*3*). Our results support the notion that the low-relief landscape atop the eastern Tibetan Plateau was elevated before Miocene time and that basin integration and headward incision into this high-standing plateau drive rapid incision downstream of this former topographic divide. Progressive captures and integration of sub-basins could have driven punctuated episodes of rapid incision as drainage divides were breached (*25*), although these events are not yet evident at the scale of this study. Future work to quantify the timing of breaching of drainage boundaries and the history of Miocene lake recession (*27*) will perhaps resolve the linkage between river incision and exhumation along the eastern margin of the plateau (*34*). Regardless, our study suggests that the pace and tempo of fluvial incision in response to plateau uplift have a rich and complicated history that involves substantial lag times between uplift and incision.

## MATERIALS AND METHODS

### 1D numerical modeling of Yangtze incision

We use the 1D stream-power incision model (SPIM) to simulate the river incision history along the Yangtze River. According to the SPIM, the rate of change in river bed elevation is a function of uplift (*U*) and incision

$$\frac{dz(x)}{dt}=U-KA{\left(x\right)}^{m}{\left(\frac{dz(x)}{dx}\right)}^{n}$$ (1)

where *x* is the horizontal distance along the river, *K* is the bedrock erodibility, *A* is upstream area, and *dz*/*dx* is the river gradient. *m* and *n* are exponents that reflect the hydrology, river width, and mechanisms of incision (*47*). We study the Yangtze River, with a catchment area of 3.6 × 10^6^ km^2^, from the drainage divide on the Tibetan Plateau to the confluence of the Min Jiang/Dadu River with the Yangtze watershed in the Sichuan Basin. The Sichuan Basin is therefore our base level and represents our uplift boundary. We performed two SPIM runs to quantify the effect of adding upstream drainage area to a synthetic steady-state river profile of the Yangtze River and their impact on the amount of river incision (fig. S1). We call the steady-state profile “synthetic” because our SPIM does not capture or represent the tectonic uplift history of the eastern Tibetan Plateau before 15 Ma ago, but instead we focus on obtaining a steady-state river profile that best matches the conditions within the river starting at 15 Ma ago to test only the effect of drainage integration to downstream incision.

The spatial resolution (Δ*x*) of the model was fixed to 5000 m, and the time-step length Δ*t* was set to 375,000 years. Slope area plots yielded a river concavity θ *= m*/*n* of 0.32 for the upper Yangtze, and assuming a linear kinematic wave model for river incision (*n* = 1), we adopt a value of *m* = 0.32 and a value of 10^−6^ m^0.36^ year^−1^ for *K* in our simulations. Values of *K* were determined in a trial and error approach by manually adjusting the value of *K* to best match the river profile’s present-day condition. The result matches well-reported *K* values within the Yangtze and other rivers worldwide that range between 10^−4^ and 10^−6^ m^0.36^ year^−1^ (*48*). The uplift was uniform over the entire synthetic steady-state profile and was set to a minimal background uplift rate of 60 m/Ma that matches slow modern GPS rates (*49*) and agrees with long-term plateau-wide exhumation rates (*21*). A steady-state profile was reached after a runtime of 65 Ma. At that time, the headwaters, i.e., the low-relief high-elevation surface, reached an elevation of 4200 m in the model run that corresponds to the conditions present around 15 Ma ago at the start of the drainage basin expansion.

We assume two scenarios in terms of how much upstream drainage area was added to the Yangtze after 15 Ma: (i) We added an upstream drainage area of 0.85 × 10^6^ m^2^ to the steady-state river profile, i.e., the extent of Miocene lake sediments found today in the upper Yangtze catchment (*27*); and (ii) we added an upstream-drainage area of 1.36 × 10^6^ m^2^ to the steady-state river profile, i.e., the combined drainage area of the extent of Miocene lake sediments and meandering river stretches (Fig. 1A and fig. S1). The added drainage area in the two SPIM runs is best expressed as a quadratic function of distance following Hack’s law that matched the observed total added drainage basin area after 15 Ma ago (*50*). The uplift rate over the catchment remained unchanged over a model runtime of 15 Ma at 60 m/Ma (*49*) to quantify the effect of upstream drainage area expansion. Each scenario ran for 15 Ma after the upstream river network area was instantaneously added, which represent the time between the cessation of Miocene lacustrine deposition (*27*) and the present-day condition. The numerical implementation of the SPIM relies on a total variation diminishing finite volume method (TVD-FVM), which has been extensively applied to study not only dam break problems but also in river geomorphology, and soil erosion and coupled sediment transport (*36*). The main advantage of the TVD-FVM is that it better captures and conserves discontinuities such as knickpoints.

### 2D numerical modeling of spatial distribution of knickpoints

We explore two conceptual models of topographic development in the upper Yangtze catchment using the 2D TTLEM (*37*). In the first model, knickpoints in longitudinal profiles of the Yangtze and its tributaries are related to a fall in base level at some location along the Yangtze River. In this case, the change in relative baselevel will generate a knickpoint that migrates upstream along the main river and then into tributaries (*40*, *51*, *52*). The second model reflects our hypothesis of upstream drainage basin integration. An instantaneous increase in drainage area would lead to a transient state of higher incision rates as the river adjusts its longitudinal profile to the consequent increase of runoff and stream power. Predicated on the SPIM (*47*), fluvial incision *E* is the product of drainage area *A* and along-river gradient (*dz*/*dx*) raised to the power exponents *m* and *n*. Drainage basin integration means that processes such as river piracy or overspill from an adjacent basin will suddenly add drainage area *A*_int_ at some location and downstream

$$E(x)=K{\left[A(x)+A_{\mathrm{int}}(x)\right]}^{m}{\left(\frac{dz(x)}{dx}\right)}^{n}$$ (2)

Although the model is formulated in 2D, the *x* in Eq. 1 is the quasi-1D distance in the tree network that represents the flow network on the simulated surface. Depending on the gained area, the drainage basin integration upstream of a trunk stream would lead to higher incision rates along the trunk stream, particularly in its upper reaches where the relative growth in drainage area is largest. At confluences, lowering of the trunk stream would then simultaneously generate a series of knickpoints that migrate upstream into the tributaries, albeit at rates dictated by the upstream areas of the tributaries. Because of this effect, the base-level fall/change in uplift rate model generates different spatial patterns of knickpoints than the drainage basin integration model that may be diagnosed by their network position (see text for discussion).

To explore the spatial patterns of knickpoints associated with the two conceptual models, we used TTLEM (*37*). TTLEM is a numerical simulation tool that solves the SPIM and hillslope diffusion model. We generated a random surface on a rectangular grid with a spatial resolution of 50 m and an extent of 100 km by 25 km. Slightly tilting the surface forced flow to the lower left corner of the grid. Tectonic uplift is uniform at a rate of 0.01 Ma^−1^ except at the lower left corner where uplift is zero. We set the diffusivity to zero so that fluvial incision is the only landscape forming process. Exponents in the SPIM were set to *m* = 0.5 and *n* = 1, and the erosivity was *K* = 4 × 10^−5^ year^−1^. We used the FASTSCAPE algorithm, an implicit finite difference scheme (*53*), to solve the SPIM and ran the model until the landscape evolution reached a steady state between uplift and erosion (fig. S2). Subsequently, the steady-state surface served as initial conditions for two different simulations. The first simulation involved an instantaneous base-level fall of 500 m at the lower left corner of the DEM, thus mimicking our first conceptual model. In the second simulation, we added 2000-km^2^ upstream area to the right upper corner. Again, we ran both simulations until steady state, yet this time recording surfaces at various time steps, as we were primarily interested in the transient stages of the development of longitudinal river profiles.

We analyzed the simulated transient surfaces by extracting stream networks and identifying knickpoints as described above. Moreover, we calculated χ maps of the simulated river networks and extracted χ values at knickpoint locations. χ values are derived from the transformation of the horizontal coordinate measured as distance from the outlet by the integral approach (χ analysis) (*40*). The transformation entails those transient erosional features such as knickpoints that originated from a common source plot at the same value of χ (*40*). Figure 3 shows the location of knickpoints in χ space for the two different models. Base-level fall generates a series of knickpoints with narrow, unimodal χ distributions that migrate to higher values of χ as model time proceeds (Fig. 3A). In the model, slight deviations from perfectly unimodal distributions arise from numerical diffusion. Drainage basin integration, in turn, creates multimodal distributions of χ if χ transformation derives from integration from the outlet (Fig. 3B). However, if we calculate χ by integration from the trunk river, knickpoints exhibit unimodal frequency distributions (Fig. 3C). We thus propose that the unimodality of knickpoint χ values measured both from a single location along the trunk river and from multiple tributary confluences along the trunk river allows us to test the two competing hypotheses: incision by base-level fall/change in uplift rate and incision by drainage basin integration.

### Knickpoint analysis in the upper Yangtze River basin

To analyze the two models for the Yangtze River, we derived the stream network for a minimum supporting catchment area of 5 km^2^ from Shuttle Radar Topography Mission 3 (90-m spatial resolution) Digital Elevation Model in TopoToolbox (fig. S3). We identify knickpoints along river profiles using TopoToolbox (*54*) and the function knickpointfinder (*55*). The function iteratively adjusts a strictly upward concave profile $\hat{z}$ to the actual profile until the maximum difference *d*_max_ between *z*_min_ and $\hat{z}$ is less than the tolerance offset *d*_tol_ (fig. S4). We ran knickpointfinder with a tolerance *d*_tol_ = 250 m (see fig. S4). We then calculated two χ transformations of the river network—one sourced at the outlet of our stream network and the other sourced at the Yangtze River. From the two different χ maps, we extracted the χ values for all knickpoints (χ_kp_). To estimate the spatial dependence of knickpoints on χ, we used a nonparametric approach to investigate spatial point patterns (*56*) and adapted it to the geometry of river networks (*57*, *58*). The approach uses a kernel smoothing function to estimate the spatial density of points while normalizing for the abundance of nodes in the river network with values of the covariates. Confidence intervals around these estimates were obtained using 1000 bootstrap samples (Fig. 4, C and D, and fig. S3). As the resulting knickpoint χ values show a more peaked, unimodal distribution (Fig. 4, C and D), we infer that drainage basin integration is an equally probable, if not more probable, explanation for our observed distribution of knickpoints within the Yangtze given our assumptions about the mechanisms and velocities of knickpoint migration.

## Supplementary Materials

### This PDF file includes:

Figs. S1 to S4 References
