# Supplementary material for: Accelerated Miocene incision along the Yangtze River driven by headward drainage basin expansion
Source: Sci Adv. 2023 Sep 8;9(36):eadh1636. doi: 10.1126/sciadv.adh1636 (PMC10491212; doi:10.1126/sciadv.adh1636)
Supplement: Supplementary file 1 — Figs. S1 to S4 References [file sciadv.adh1636_sm.pdf]

Science Advances
AAAS

Supplementary Materials for

Accelerated Miocene incision along the Yangtze River driven by headward drainage basin expansion

Alexander Rohrmann *et al*.

Corresponding author: Alexander Rohrmann, alexander.rohrmann@fu-berlin.de

Sci. Adv. **9**, eadh1636 (2023)
DOI: 10.1126/sciadv.adh1636

This PDF file includes:

Figs. S1 to S4
References

Supplementary Materials

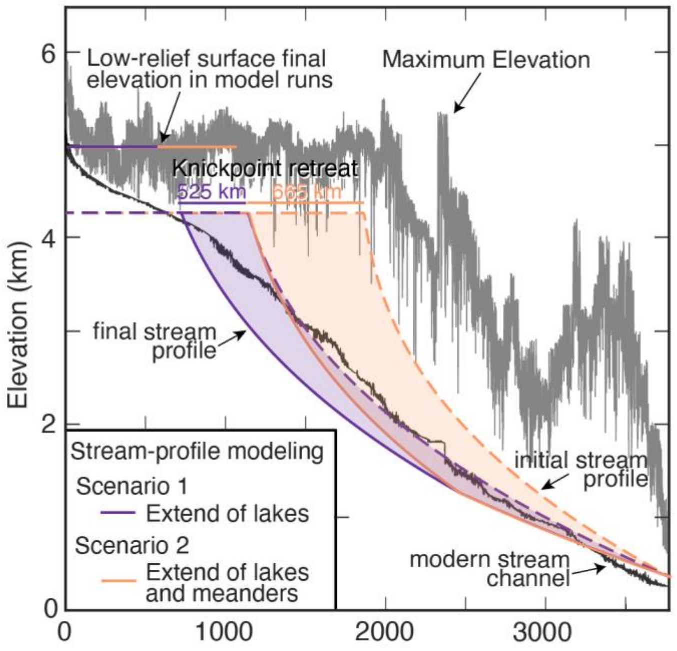

Fig. S1. 1D LEM of the Yangtze River. The two scenarios (purple: extent of lakes; orange: extent of lakes and meanders) modeled with the TVD-FVM (*36*). The dashed lines indicate the initial stream-profile before the upstream area was added and the final stream-profile is shown as a solid line. In addition, the 40 km minimum and maximum elevation swath-profile along the Yangtze is shown.

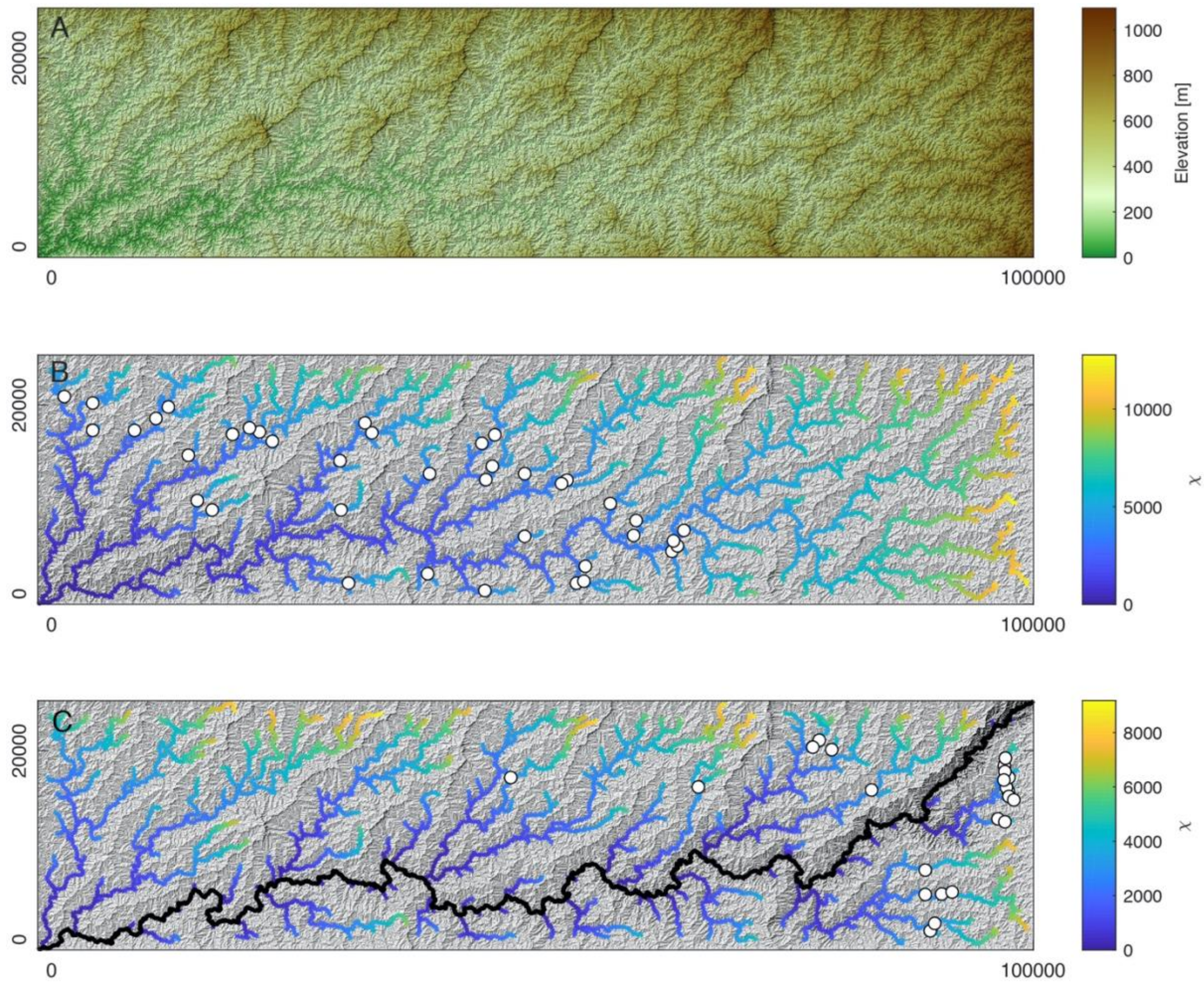

Fig. S2. Simulated transient responses to base-level fall and drainage basin integration. (**A**) Simulated steady-state surface as initial surface to subsequent model runs. (**B**) Chi-values and spatial distribution of knickpoints following base-level fall at the outlet (lower left corner). (**C**) χ-values of the tributaries and spatial distribution of knickpoints following drainage basin integration at the upper right corner. The χ-values in the tributaries are measured from the confluence with the trunk river.

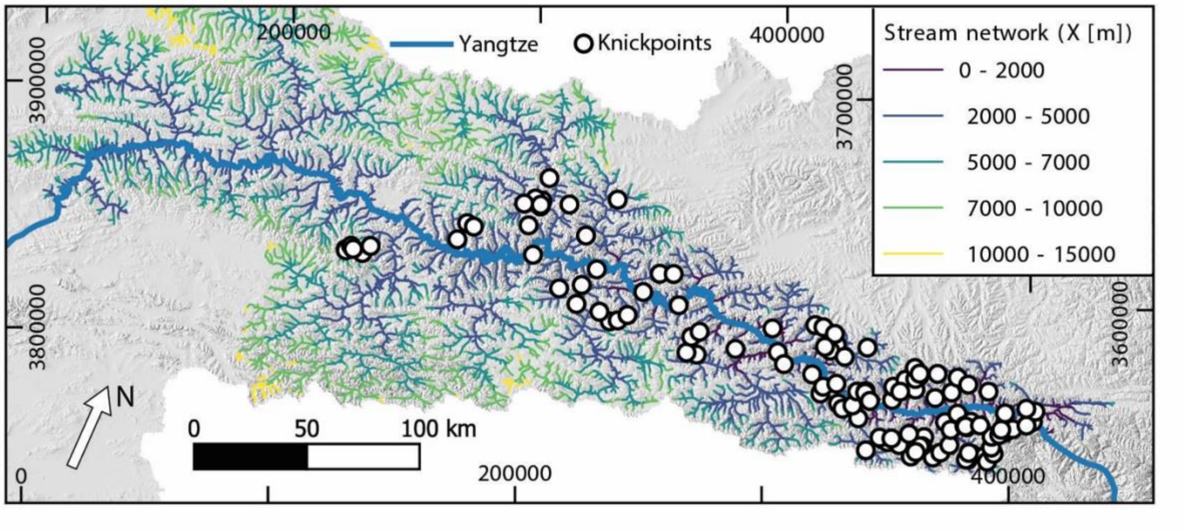

Fig. S3. Map of upper Yangtze catchment chi-values and knickpoints. The function knickpointfinder was used to identify knickpoints with a tolerance d_{tol} = 50 m.

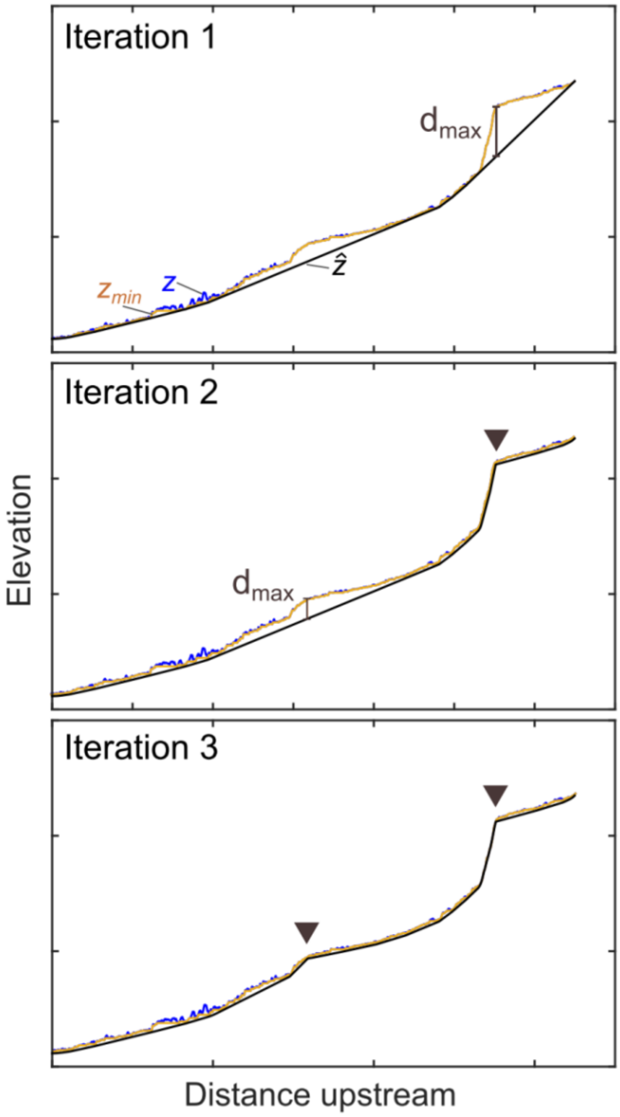

Fig. S4. Automated knickpoint identification. Automated knickpoint identification by iteratively separating concave upward stream sections with convex knickpoints (black triangles).

REFERENCES AND NOTES

1. W. M. Davis, The Geographical cycle. *Geogr. J.* **14**, 481–504 (1899).

2. P. van der Beek, J. van Melle, S. Guillot, A. Pêcher, P. W. Reiners, S. Nicolescu, M. Latif, Eocene Tibetan plateau remnants preserved in the northwest Himalaya. *Nat. Geosci.* **2**, 364–368 (2009).

3. M. K. Clark, L. H. Royden, K. X. Whipple, B. C. Burchfiel, X. Zhang, W. Tang, Use of a regional, relict landscape to measure vertical deformation of the eastern Tibetan Plateau. *J. Geophys. Res.* **111**, 10.1029/2005JF000294 (2006).

4. C. W. Wobus, G. E. Tucker, R. S. Anderson, Does climate change create distinctive patterns of landscape incision? *J. Geophys. Res.* **115**, 10.1029/2009JF001562 (2010).

5. R. O. Lease, T. A. Ehlers, Incision into the Eastern Andean Plateau During Pliocene Cooling. *Science* **341**, 774–776 (2013).

6. L. E. Hasbargen, C. Paola, Landscape instability in an experimental drainage basin. *Geology* **28**, 1067–1070 (2000).

7. S. D. Willett, S. W. Mccoy, J. T. Perron, L. Goren, C.-Y. Chen, Dynamic reorganization of River Basins. *Science* **343**, 1248765 (2014)..

8. K. X. Whipple, A. M. Forte, R. A. DiBiase, N. M. Gasparini, W. B. Ouimet, Timescales of landscape response to divide migration and drainage capture: Implications for the role of divide mobility in landscape evolution. *J. Geophys. Res. Earth* **122**, 248–273 (2017).

9. W. B. Ouimet, K. X. Whipple, L. H. Royden, Z. Sun, Z. Chen, The influence of large landslides on river incision in a transient landscape: Eastern margin of the Tibetan Plateau (Sichuan, China). *Geol. Soc. Am. Bull.* **119**, 1462–1476 (2007).

10. S. F. Gallen, Lithologic controls on landscape dynamics and aquatic species evolution in post-orogenic mountains. *Earth Planet. Sci. Lett.* **493**, 150–160 (2018).

11. M. K. Clark, L. M. Schoenbohm, L. H. Royden, K. X. Whipple, B. C. Burchfiel, X. Zhang, W. Tang, E. Wang, L. Chen, Surface uplift, tectonics, and erosion of eastern Tibet from large-scale drainage patterns. *Tectonics* **23**, 10.1029/2002TC001402 (2004).

12. E. Kirby, P. W. Reiners, M. A. Krol, K. X. Whipple, K. V. Hodges, K. A. Farley, Late Cenozoic evolution of the eastern margin of the Tibetan Plateau: Inferences from $^{40}Ar/^{39}Ar$ and (U-Th)/He thermochronology. *Tectonics* **21**, 1-1-1-20 (2002).

13. K. Cao, Y. Tian, P. van der Beek, G. Wang, T. Shen, P. Reiners, M. Bernet, L. Husson, Southwestward growth of plateau surfaces in eastern Tibet. *Earth Sci. Rev.* **232**, 104160 (2022).

14. W. Ouimet, K. Whipple, L. Royden, P. Reiners, K. Hodges, M. Pringle, Regional incision of the eastern margin of the Tibetan Plateau. *Lithosphere* **2**, 50–63 (2010).

15. R. Yang, S. D. Willett, L. Goren, In situ low-relief landscape formation as a result of river network disruption. *Nature* **520**, 526–529 (2015).

16. K. X. Whipple, R. A. Dibiase, W. B. Ouimet, A. M. Forte, Preservation or piracy: Diagnosing low-relief, high-elevation surface formation mechanisms. *Geology* **45**, 91–94 (2017).

17. R. Yang, M. G. Fellin, F. Herman, S. D. Willett, W. Wang, C. Maden, Spatial and temporal pattern of erosion in the Three Rivers Region, southeastern Tibet. *Earth Planet. Sci. Lett.* **433**, 10–20 (2016).

18. M. Fox, A. Carter, J.-G. Dai, How continuous are the "Relict" landscapes of Southeastern Tibet? *Front. Earth Sci.* **8**, 587597 (2020).

19. X. P. Yuan, K. L. Huppert, J. Braun, X. Shen, J. Liu-Zeng, L. Guerit, S. G. Wolf, J. F. Zhang, M. Jolivet, Propagating uplift controls on high-elevation, low-relief landscape formation in the southeast Tibetan Plateau. *Geology* **50**, 60–65 (2022).

20. G. D. Hoke, J. Liu-Zeng, M. T. Hren, G. K. Wissink, C. N. Garzione, Stable isotopes reveal high southeast Tibetan Plateau margin since the Paleogene. *Earth Planet. Sci. Lett.* **394**, 270–278 (2014).

21. A. Rohrmann, P. Kapp, B. Carrapa, P. W. Reiners, J. Guynn, L. Ding, M. Heizler, Thermochronologic evidence for plateau formation in central Tibet by 45 Ma. *Geology* **40**, 187–190 (2012).

22. J. Nie, G. Ruetenik, K. Gallagher, G. Hoke, C. N. Garzione, W. Wang, D. Stockli, X. Hu, Z. Wang, Y. Wang, T. Stevens, M. Danišík, S. Liu, Rapid incision of the Mekong River in the middle Miocene linked to monsoonal precipitation. *Nat. Geosci.* **11**, 944–948 (2018).

23. A. Replumaz, M. San José, A. Margirier, P. Beek, C. Gautheron, P. H. Leloup, X. Ou, C. Kai, G. C. Wang, Y. Z. Zhang, P. G. Valla, M. Balvay, Tectonic control on rapid late Miocene—Quaternary incision of the Mekong River Knickzone, southeast Tibetan Plateau. *Tectonics* **39**, e2019TC005782 (2020).

24. X. Shen, J. Braun, X. Yuan, Southeastern margin of the Tibetan Plateau stopped expanding in the late Miocene. *Earth Planet. Sci. Lett.* **583**, 117446 (2022).

25. W. H. Craddock, E. Kirby, N. W. Harkins, H. Zhang, X. Shi, J. Liu, Rapid fluvial incision along the Yellow River during headward basin integration. *Nat. Geosci.* **3**, 209–213 (2010).

26. E. Kirby, K. X. Whipple, Expression of active tectonics in erosional landscapes. *J. Struct. Geol.* **44**, 54–75 (2012).

27. W. Zhenhan, P. J. Barosh, W. Zhonghai, H. Daogong, Z. Xun, Y. Peisheng, Vast early Miocene lakes of the central Tibetan Plateau. *Geol. Soc. Am. Bull.* **120**, 1326–1337 (2008).

28. D. B. Rowley, B. S. Currie, Palaeo-altimetry of the late Eocene to Miocene Lunpola basin, central Tibet. *Nature* **439**, 677–681 (2006).

29. L. M. Staisch, N. A. Niemi, M. K. Clark, H. Chang, Eocene to late Oligocene history of crustal shortening within the Hoh Xil Basin and implications for the uplift history of the northern Tibetan Plateau. *Tectonics* **35**, 862–895 (2016).

30. P. J. Polissar, K. H. Freeman, D. B. Rowley, F. A. McInerney, B. S. Currie, Paleoaltimetry of the Tibetan Plateau from D/H ratios of lipid biomarkers. *Earth Planet. Sci. Lett.* **287**, 64–76 (2009).

31. B. Sun, Y. F. Wang, C. S. Li, J. Yang, J. F. Li, Y. L. Li, T. Deng, S. Q. Wang, M. Zhao, R. A. Spicer, D. K. Ferguson, R. C. Mehrotra, Early Miocene elevation in northern Tibet estimated by palaeobotanical evidence. *Sci. Rep.* **5**, 10379 (2015).

32. B. Hallet, P. Molnar, Distorted drainage basins as markers of crustal strain east of the Himalaya. *J. Geophys. Res.* **106**, 13697–13709 (2001).

33. W. A. Tarr, Intrenched and Incised Meanders of Some Streams on the Northern Slope of the Ozark Plateau in Missouri. *J. Geol.* **32**, 583–600 (1924).

34. H. Zhang, M. E. Oskin, J. Liu-zeng, P. Zhang, P. W. Reiners, P. Xiao, Pulsed exhumation of interior eastern Tibet: Implications for relief generation mechanisms and the origin of high-elevation planation surfaces. *Earth Planet. Sci. Lett.* **449**, 176–185 (2016).

35. E. Wang, E. Kirby, K.P. Furlong, M. van Soest, G. Xu, X. Shi, P. J. J. Kamp, K. V. Hodges, Two-phase growth of high topography in eastern Tibet during the Cenozoic. *Nat. Geosci.* **5**, 640–645 (2012).

36. B. Campforts, G. Govers, Keeping the edge: A numerical method that avoids knickpoint smearing when solving the stream power law. *J. Geophys. Res. Earth Surf.* **120**, 1189–1205 (2015).

37. B. Campforts, W. Schwanghart, G. Govers, Accurate simulation of transient landscape evolution by eliminating numerical diffusion: The TTLEM 1.0 model *Earth Surf. Dyn.* **5**, 47–66 (2017).

38. R. A. DiBiase, K. X. Whipple, M. P. Lamb, A. M. Heimsath, The role of waterfalls and knickzones in controlling the style and pace of landscape adjustment in the western San Gabriel Mountains, California. *Geol. Soc. Am. Bull.* **127**, 539–559 (2015).

39. I. Haviv, Y. Enzel, K. X. Whipple, E. Zilberman, J. Stone, A. Matmon, L. K. Fifield, Amplified erosion above waterfalls and oversteepened bedrock reaches. *J. Geophys. Res. Earth Surf.* **111**, 10.1029/2006JF000461 (2006).

40. J. T. Perron, L. Royden, An integral approach to bedrock river profile analysis. *Earth Surf. Process. Landf.* **38**, 570–576 (2013).

41. D. McPhillips, G. D. Hoke, J. Liu-Zeng, P. R. Bierman, D. H. Rood, S. Niedermann, Dating the incision of the Yangtze River gorge at the First Bend using three-nuclide burial ages. *Geophys. Res. Lett.* **43**, 101–110 (2016).

42. A. R. Duvall, M. K. Clark, B. Avdeev, K. A. Farley, Z. Chen, Widespread late Cenozoic increase in erosion rates across the interior of eastern Tibet constrained by detrital low-temperature thermochronometry. *Tectonics* **31**, 10.1029/2011TC002969 (2012).

43. P. Kong, Y. Zheng, M. W. Caffee, Provenance and time constraints on the formation of the first bend of the Yangtze River. *Geochem. Geophys. Geosyst.* **13**, 10.1029/2012GC004140 (2012).

44. H. Zheng, P. D. Clift, P. Wang, R. Tada, J. Jia, M. He, F. Jourdan, Pre-miocene birth of the Yangtze River. *Proc. Natl. Acad. Sci. U.S.A.* **110**, 7556–7561 (2013).

45. Z. Zhang, J. S. Daly, Y. Tian, S. Tyrrell, X. Sun, E. Badenszki, Y. Qin, Z. Cheng, R. Guo, Sedimentary provenance perspectives on the evolution of the major rivers draining the eastern Tibetan Plateau. *Earth Sci. Rev.* **232**, 104151 (2022).

46. R. Guo, X. Sun, C. Li, Y. Li, C. Wei, Z. Zhang, Y. Leng, U. Klötzli, G. Li, L. Lv, X. Chen, Cenozoic evolution of the Yangtze River: Constraints from detrital zircon U-Pb ages. *Palaeogeogr. Palaeoclimatol. Palaeoecol.* **579**, 110586 (2021).

47. D. Lague, The stream power river incision model: Evidence, theory and beyond. *Earth Surf. Process. Landf.* **39**, 38–61 (2014).

48. J. L. Schmidt, P. K. Zeitler, F. J. Pazzaglia, M. M. Tremblay, D. L. Shuster, M. Fox, Knickpoint evolution on the Yarlung river: Evidence for late Cenozoic uplift of the southeastern Tibetan plateau margin. *Earth Planet. Sci. Lett.* **430**, 448–457 (2015).

49. S. Liang, W. Gan, C. Shen, G. Xiao, J. Liu, W. Chen, X. Ding, D. Zhou, Three-dimensional velocity field of present-day crustal motion of the Tibetan Plateau derived from GPS measurements. *J. Geophys. Res. Solid Earth* **118**, 5722–5732 (2013).

50. J. T. Hack, *Studies of longitudinal stream profiles in Virginia and Maryland* (U.S. Government Printing Office, 1957), vol. 294.

51. G. E. Tucker, K. X. Whipple, Topographic outcomes predicted by stream erosion models: Sensitivity analysis and intermodel comparison. *J. Geophys. Res.* **107**, ETG 1-1-ETG 1-16 (2002).

52. M. M. Berlin, R. S. Anderson, Modeling of knickpoint retreat on the Roan Plateau, western Colorado. *J. Geophys. Res.* **112**, 10.1029/2006JF000553 (2007).

53. J. Braun, S. D. Willett, A very efficient *O*(*n*), implicit and parallel method to solve the stream power equation governing fluvial incision and landscape evolution. *Geomorphology* **180-181**, 170–179 (2013).

54. W. Schwanghart, D. Scherler, Short Communication: TopoToolbox 2 – MATLAB-based software for topographic analysis and modeling in Earth surface sciences. *Earth Surf. Dyn.* **2**, 1–7 (2014).

55. A. Stolle, W. Schwanghart, C. Andermann, A. Bernhardt, M. Fort, J. D. Jansen, Protracted river response to medieval earthquakes. *Earth Surf. Process. Landf.* **44**, 331–341 (2019).

56. A. Baddeley, Y.-M. Chang, Y. Song, R. Turner, Nonparametric estimation of the dependence of a spatial point process on spatial covariates. *Stat. Interface* **5**, 221–236 (2012).

57. G. McSwiggan, A. Baddeley, G. Nair, Kernel Density Estimation on a Linear Network. *Scand. J. Stat.* **44**, 324–345 (2017).

58. W. Schwanghart, C. Molkenthin, D. Scherler, A systematic approach and software for the analysis of point patterns on river networks. *Earth Surf. Process. Landf.* **46**, 1847–1862 (2021).
